# Developing and Testing the Validity and Reliability of the Brief Adolescent Respiratory System Health Assessment Scale-Student Version in a Chinese Sample

**DOI:** 10.3389/fped.2021.713066

**Published:** 2021-08-17

**Authors:** Lingwei Tao, Yana Gao, Hongzhe Dou, Xuekun Wu, Lu Yan, Danyang Liu, Yuejia Zhao, Qingchun Zhao, Peiyu Wang, Yumei Zhang

**Affiliations:** ^1^School of Public Health, Peking University Health Science Center, Beijing, China; ^2^Obstetrics Department, Affiliated Hospital of Hebei University, Baoding, China; ^3^Blood Transfusion Department, Affiliated Hospital of Hebei University, Baoding, China; ^4^Outpatient Department, Affiliated Hospital of Hebei University, Baoding, China; ^5^Blood Sampling Center, Affiliated Hospital of Hebei University, Baoding, China; ^6^Orthopedics Department, Affiliated Hospital of Hebei University, Baoding, China; ^7^Department of Anesthesiology, Affiliated Hospital of Hebei University, Baoding, China

**Keywords:** adolescent, respiratory system, health status, validity, reliability

## Abstract

**Objectives:** To develop a Brief Adolescent Respiratory System Health Assessment Scale-Student Version (BARSHAS-SV) and test the validity and reliability of the scale.

**Methods:** Considering common respiratory system diseases and respiratory system symptoms as a theoretical basis, researchers developed a Brief Adolescent Respiratory System Health Assessment Scale-Student Version-I (BARSHAS-SV-I). After six medical experts reviewed the BARSHAS-SV-I, and six adolescents tested the BARSHAS-SV-I, researchers developed an updated BARSHAS-SV-II. Researchers randomly selected two middle schools in Baoding, China. Thousand twenty nine valid questionnaires were recovered. Researchers evaluated the validity and reliability of the scale and obtained the final version of the scale (BARSHAS-SV). The exploratory factor analysis (EFA) and the confirmatory factor analysis (CFA) were used to evaluate the construct validity of the scale. The content validity index (CVI) was used to evaluate the content validity of the scale. The Cronbach's α coefficient and the mean inter-item correlation coefficient (MIIC) were used to assess the reliability of the scale.

**Results:** BARSHAS-SV Cronbach's α = 0.910, content validity = 0.941, and factor cumulative variance contribution rate = 64.047% conducting EFA. Conducting CFA, Chi square value (χ2) = 233.806, degrees of freedom (df) = 106, Chi square value/degree of freedom (χ2/df) = 2.206, root-mean-square error of approximation (RMSEA) = 0.063, normed fit index (NFI) = 0.922, goodness of fit index (GFI) = 0.917, Tueker-Lewis index (TLI) = 0.942, comparative fit index (CFI) = 0.955, incremental fit index (IFI) = 0.956. BARSHAS-SV consisted of 4 dimensions and 17 items. Four factors were as follows: Factor 1, mild respiratory system diseases (Cronbach's α coefficient = 0.781); Factor 2, severe respiratory system diseases (Cronbach's α coefficient = 0.829); Factor 3, respiratory system symptoms (Cronbach's α coefficient = 0.835); Factor 4, treatment and recovery of respiratory system diseases (Cronbach's α coefficient = 0.845).

**Conclusions:** BARSHAS-SV is a valid and reliable method that can be applied to assess adolescent respiratory system health status. BARSHAS-SV may help teachers and medical staff in schools to quickly and conveniently evaluate the adolescent respiratory system health status and identify respiratory issues.

## Introduction

The prevalence of respiratory system diseases in adolescents is increasing worldwide (1). Acute infectious diseases, which are the most common issue, are a threat to adolescent health (2). For example, coronavirus disease 2019 (COVID-19), caused by severe acute respiratory syndrome coronavirus 2 (SARS-CoV-2), is a global pandemic of respiratory system (3). As schools begin to reopen, it becomes clear that SARS-CoV-2 infection may cause serious health consequences among adolescents (4). In addition, chronic diseases are also a concern. One of the most prevalent respiratory health conditions in adolescents is chronic respiratory disease (5). In adolescents, chronic respiratory disease can lead to a reduced pubertal growth spurt and delayed onset of puberty (6). Overall, respiratory diseases have a negative impact on pulmonary function, potentially resulting in its early deterioration (1). With the increase of atmospheric concentrations of nitrogen oxides, sulfur dioxide, carbon monoxide, and suspended particles, the variation of pathogenic microorganisms, and the increase of drug-resistant bacteria, more and more adolescents suffer from respiratory system diseases (2, 7, 8). Adolescent respiratory system diseases remain a major challenge for global health (9, 10). This is a serious public health problem that affects the adolescent group, and full understanding of the adolescent respiratory system health status is especially important.

By carrying out intervention programs for respiratory system diseases as well as creating an effective method that evaluates adolescent respiratory system health status, we can reduce disease symptoms, increase aerobic fitness and physical strength, improve pulmonary function, and enhance the quality of life among adolescents (11). Measurement of respiratory system health status is very important for the evaluation of physical development in the adolescents. However, there is currently a lack of a scale specifically designed to assess the health status of the respiratory system of adolescents in schools. Therefore, the research team developed a Brief Adolescent Respiratory System Health Assessment Scale-Student Version (BARSHAS-SV) and tested its validity and reliability.

## Methods

### Development of BARSHAS-SV-I

Considering common respiratory system diseases and respiratory system symptoms as a theoretical basis (2), in addition to investigating extensive literature references, the research team developed an initial scale (Brief Adolescent Respiratory System Health Assessment Scale-Student Version-I, BARSHAS-SV-I). This initial scale included a total of 20 items and 3 dimensions. The research team then named the three dimensions as follows: Dimension 1, common respiratory system diseases; Dimension 2, respiratory system symptoms; Dimension 3, treatment and recovery of respiratory system diseases. The items of BARSHAS-SV-I were presented in a way that made them easy to understand for the adolescent population (12, 13).

### Development of BARSHAS-SV-II

Six medical experts (two clinical doctors, two health care experts, and two clinical nurses) were invited to evaluate the scale content validity. The evaluation standard, which was used to evaluate the scale content validity ranged from 3 (strongly related) to 1 (not related). According to expert feedback, 3 items in the BARSHAS-SV-I were removed from the initial scale, which resulted in an updated scale (BARSHAS-SV-II). The updated scale (BARSHAS-SV-II) included a total of 17 items and 3 dimensions. The names of the dimensions in the BARSHAS-SV-II remained unchanged. BARSHAS-SV-IIused the Likert 5-point method (disagree = 1; agree a small part = 2; moderately agree = 3; agree most = 4; completely agree = 5). In reverse scoring, the scores were 5 points, 4 points, 3 points, 2 points, and 1 point, respectively. The total score of the BARSHAS-SV-II was determined by the sum of all items' scores. The higher the total score of the scale, the better the adolescent respiratory system health status. Subsequently, researchers invited 6 adolescents to complete BARSHAS-SV-II in order to make the research team be able to test the comprehension of statement expressions and possibly improve the wording of the scale. All items in the BARSHAS-SV-II were presented in simple and reader-friendly language so that the adolescent subjects could easily understand the meaning of each item in the BARSHAS-SV-II (12, 13).

### Development of the Final BARSHAS-SV and Large Sample Test

**Figure 1:**
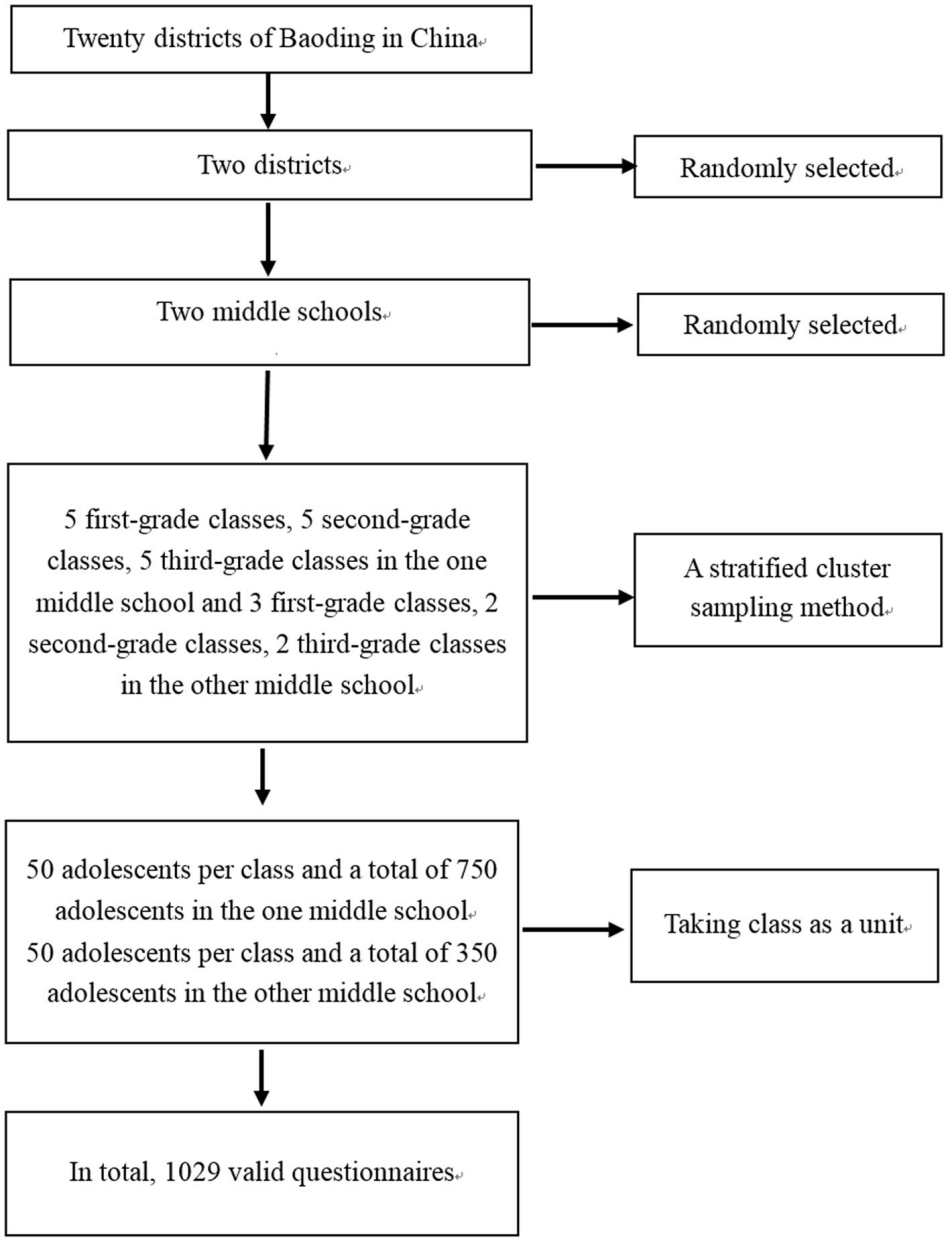
Study participant flow diagram in this study.

Researchers randomly selected two districts from the twenty districts of Baoding, China, from June 2015 to April 2016. Subsequently, two middle schools from these two districts of Baoding were randomly selected by the research team. A class was considered as a unit. By adopting a stratified cluster sampling method, researchers randomly selected five third-grade classes, five second-grade classes, and five first-grade classes from one middle school. The research team selected a total of 15 classes (50 students per class) and 750 students from the first middle school. In addition, two third-grade classes, two second-grade classes, and three first-grade classes were selected from the second middle school (a total of 350 students and 50 students per class). In this study, first grade, second grade and third grade referred to first grade, second grade and third grade only in the middle schools. A stratified cluster sampling method was the consideration of selecting five classes/grade in one middle school and 2–3 classes/grade in the other middle school. In the first middle school, there were fifteen classes for each grade. Therefore, researchers randomly selected five classes in each grade. In the second middle school, there were six classes in the third grade, six classes in the second grade, and nine classes in the first grade. Therefore, researchers randomly selected two classes in the third grade, two classes in the second grade, and three classes in the first grade. The first middle school was in the urban area, and the second middle school was in the suburbs. In total, the research team selected 1,100 adolescents. Inclusion criteria: (1) Subjects with a satisfactory capacity to comprehend questionnaires as well as answer them; (2) Subjects with no reading disabilities or no intellectual disabilities; (3) Subjects who do not suffer from any mental condition or brain diseases; and (4) Subjects who volunteered for the research. The investigators distributed 1,100 questionnaires to the adolescents. Twenty three participants did not complete demographic characteristic questionnaires or scales, and 48 participants did not complete the scale. Thus, the 71 subjects were excluded from this study. Ultimately, in this study, 1,029 valid questionnaires were returned from the subjects. After analyzing the data of 1,029 valid questionnaires from the adolescents, the research team evaluated the validity and reliability of the scale and obtained the final version of the scale (BARSHAS-SV). The flow diagram of this study is shown in Figure 1.

### Survey Method and Ethical Consideration

The investigators explained the purpose of this investigation to two middle school teaching management departments, guardians/parents of the minors, and the students themselves. After receiving consent from two middle school coordinators, guardians/parents of the minors, and the students, the investigators demonstrated to the subjects in detail how to answer these questionnaires. Standardized language and unified instruction were used in the questionnaires. The investigators conducted the study according to the Declaration of Helsinki. This study was approved by the Medical Ethics Committee of Hebei University. This study was also approved by the Health and Family Planning Commission of Hebei (NO.20150072).

### Statistical Analysis

Researchers adopted the Epidata 3.1 software to input all of the data into our computer twice, as well as to conduct a data consistency check. The data were analyzed by using the IBM SPSS Statistics 24.0 software and the AMOS 22.0 software. In this study, researchers applied descriptive statistics (medians/interquartile ranges or frequency/percentages) to explore the demographic characteristics of the adolescents. The following list shows the statistical analysis methods that the research team used for testing the validity and reliability of scale: (1) Researchers used the exploratory factor analysis (EFA) and the confirmatory factor analysis (CFA) to evaluate the construct validity of the scale. The following detailed criteria were applied for the retention of factors (14): ① Eigenvalues > 1; ② EFA scree plot; ③ Items equal to, or >2 being retained; and ④ The factor loadings > 0.5. (2) Researchers applied the content validity index (CVI) to evaluate the content validity of the scale. (3) Researchers used the Cronbach's α coefficient and the mean inter-item correlation coefficient (MIIC) to assess the reliability of the scale. The cutoff point adopted for the Cronbach's alpha coefficient was 0.70, and the cutoff point adopted for the mean inter-item correlation coefficient (MIIC) was 0.30 (12, 15). The level of significance in this study was *p* < 0.05. The model fit criteria of the scale structure are as follows (16): ① Root-mean-square error of approximation (RMSEA) <0.08; ② Chi square value/degrees of freedom (χ2 /df) <3; ③ Normed fit index (NFI) > 0.9; ④ Goodness of fit index (GFI) > 0.9; ⑤ Tueker-Lewis index (TLI) > 0.9; ⑥ Comparative fit index (CFI) > 0.9; ⑦ Incremental fit index (IFI) > 0.9.

## Results

### Characteristics of the Adolescents in the Large Sample

The research team distributed a total of 1,100 questionnaires to the adolescents in two middle schools and recovered 1,029 valid questionnaires (valid recovery rate of 93.55%). The participants included 517 males (50.24%) and 512 females (49.76%) from urban (66.57%) and rural areas (33.43%). The age of participants in this study was 14.00 (13.00, 16.00) years old (medians and interquartile ranges, IQR). Demographic characteristics of the adolescents included Han race (96.60%) and minority race (3.40%). According to monthly expenses (monthly expenses <300 yuan, 300 yuan ≤ monthly expenses <600 yuan, and monthly expenses ≥600 yuan), the adolescents were categorized into three groups, accounting for 33.43, 52.58, and 13.99%, respectively. According to the method of medical insurance, the participants were classified into three groups (urban medical insurance, new rural cooperative medical system, and self-paying), accounting for 58.02, 29.83, and 12.15%, respectively. The characteristics of the adolescents in the large sample from two middle schools are shown in Table 1.

**Table 1:** The characteristics of the adolescents in the large sample.

**Characteristics**	**Frequency/medians**	**Percentage (%)/interquartile ranges, (IQR)**
**Gender**		
Male	517	50.24%
Female	512	49.76%
Age	14.00	13.00, 16.00
**Race**		
Han	994	96.60%
Minority	35	3.40%
**Monthly expenses (yuan)**		
<300	344	33.43%
300~	541	52.58%
600~	144	13.99%
**Do you have a religious faith**		
No	955	92.81%
Yes	74	7.19%
**Place of residence**		
Urban area	685	66.57%
Rural area	344	33.43%
**Medical insurance**		
Urban medical insurance	597	58.02%
New rural cooperative medical system	307	29.83%
Self-paying	125	12.15%
**Do you live with your family**		
Yes	962	93.49%
No	67	6.51%

*The characteristics data of the large sample were presented as medians (interquartile ranges, IQR) and frequency (percentage, %)*.

### Analyses of Validity and Reliability

#### Construct Validity

**Figure 2:**
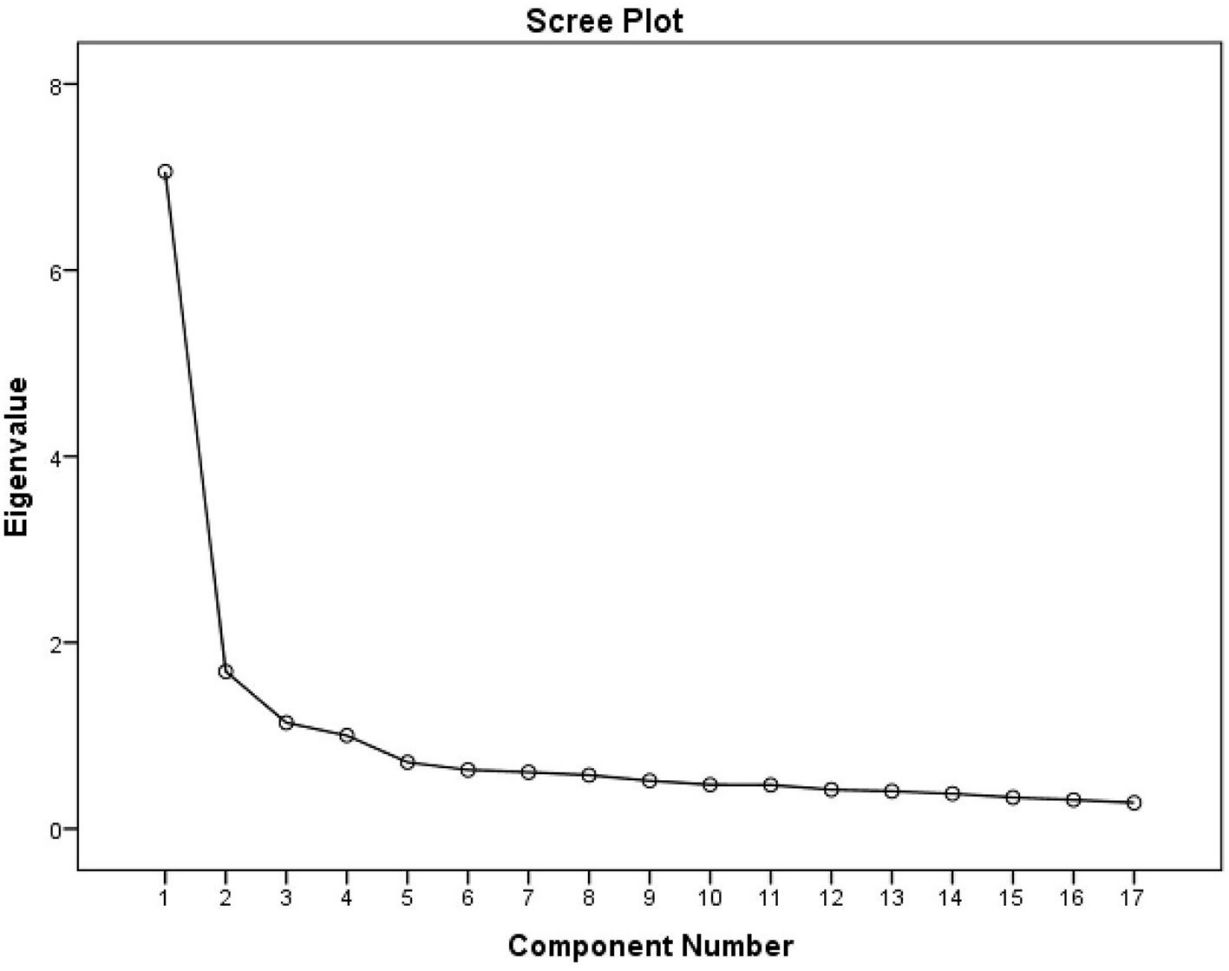
Scree plot of exploratory factor analysis.

(1) EFA. A subsample of 720 participants, randomly selected from total sample, was used in EFA. Maximum variance orthogonal rotation and principal component analysis were applied. The Bartlett sphericity test value was 5,482.205 (df = 136, *p* < 0.001), and the Kaiser-Meyer-Olkin (KMO) value was 0.924. These results revealed that the data in this study were suitable for factor analysis. Researchers conducted the factor extraction under a condition of undefined factor number. Four factors (eigenvalue greater than 1) were extracted. The cumulative variance contribution rate (%) of four factors was 64.047%. The result of the EFA scree plot also indicated that the 4-factor structure was suitable for the scale (Figure 2). Based on the aforementioned analyses, the final version of the scale consisted of 4 factors and 17 items. The research team renamed the final four factors: Factor 1, mild respiratory system diseases (three items); Factor 2, severe respiratory system diseases (five items); Factor 3, respiratory system symptoms (five items); Factor 4, treatment and recovery of respiratory system diseases (four items) (Table 2). These detailed items of the Brief Adolescent Respiratory System Health Assessment Scale-Student Version (BARSHAS-SV) are shown in Table 3, at the end of this paper.

**Table 2:** Rotated component matrix, eigenvalue, variance contribution rate and cumulative variance contribution rate.

	**Items**	**Factor 3**	**Factor 2**	**Factor 4**	**Factor 1**
	Item-1	-	-	-	0.789
	Item-2	-	-	-	0.840
	Item-3	-	-	-	0.707
	Item-4	-	0.659	-	-
	Item-5	-	0.768	-	-
	Item-6	-	0.721	-	-
	Item-7	-	0.604	-	-
	Item-8	-	0.739	-	-
	Item-9	0.644	-	-	-
	Item-10	0.712	-	-	-
	Item-11	0.685	-	-	-
	Item-12	0.738	-	-	-
	Item-13	0.630	-	-	-
	Item-14	-	-	0.723	-
	Item-15	-	-	0.750	-
	Item-16	-	-	0.744	-
	Item-17	-	-	0.678	-
Eigenvalue		3.008	2.921	2.695	2.264
Variance contribution rate (%)		17.693	17.182	15.852	13.320
Cumulative variance contribution rate (%)		17.693	34.875	50.727	64.047
Factor naming		Respiratory system symptoms	Severe respiratory system diseases	Treatment and recovery of respiratory system diseases	Mild respiratory system diseases

*Suppress absolute values <0.500. The symbol “-” showed that these values were <0.500*.

**Table 3:** Detailed items of the brief adolescent respiratory system health assessment scale-student version (BARSHAS-SV).

**Dimensions**	**Items**	**Completely agree** **5**	**Agree most** **4**	**Moderately agree** **3**	**Agree a small part** **2**	**Disagree** **1**
Mild respiratory system diseases	Item-1. I often catch a cold.					
	Item-2. I often cough.					
	Item-3. I often feel phlegm in my throat.					
Severe respiratory system diseases	Item-4. I often suffer from bronchitis.					
	Item-5. I often suffer from pneumonia.					
	Item-6. I often suffer from respiratory system injuries.					
	Item-7. I often feel weak due to respiratory system diseases.					
	Item-8. I often suffer from allergic diseases of respiratory system.					
Respiratory system symptoms	Item-9. I often have shortness of breath.					
	Item-10. I often have whistling or whooping sounds when I breathe.					
	Item-11. I often have trouble breathing when I sleep.					
	Item-12. I often walk slowly due to trouble breathing.					
	Item-13. I often have trouble breathing after I do some simple daily physical activities.					
Treatment and recovery of respiratory system diseases	Item-14. I often go to the hospital for treatments or examinations because of respiratory system diseases.					
	Item-15. I often take some medicines for respiratory system diseases.					
	Item-16. It often takes a long time for me to recover from a respiratory system disease.					
	Item-17. I often cannot study in class due to the treatment and recovery of respiratory system diseases.					

**Figure 3:**
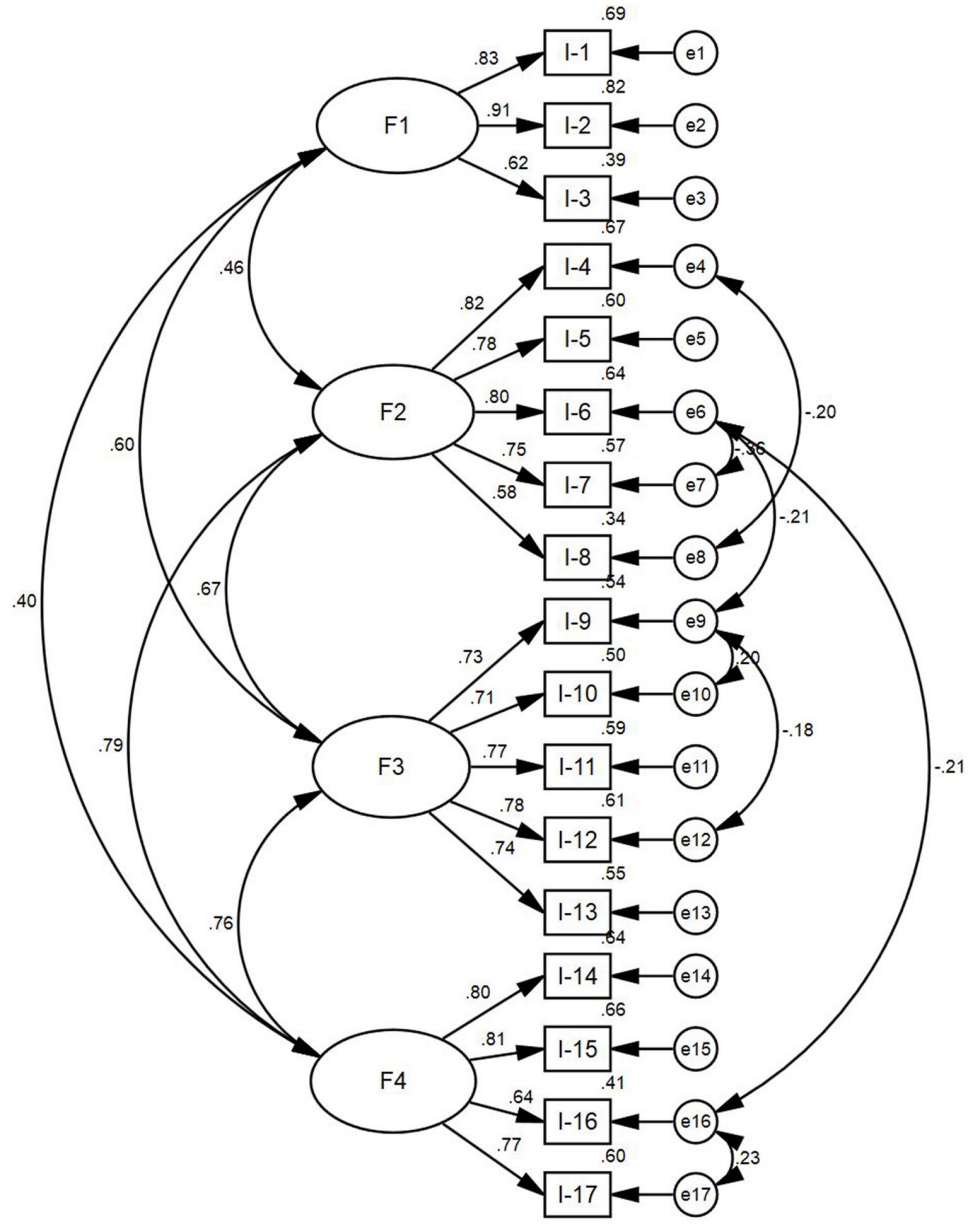
Standard path and parameter estimation of confirmatory factor analysis. F1, Factor 1, mild respiratory system diseases; F2, Factor 2, severe respiratory system diseases; F3, Factor 3, respiratory system symptoms; F4, Factor 4, treatment and recovery of respiratory system diseases.

(2) CFA. To confirm the dimensional structure found in the EFA, the researchers used the remaining 30% of sample (309 participants) and adopted the Maximum Likelihood (ML) method to perform the CFA. The results are shown in Table 4. The 4-factor structure showed a good fit to the subsample after allowing covariances between the residuals. Figure 3 shows the fitted structure. Additionally, the researchers also verified the fit of the 3-factor structure for the subsample (see Table 4). This structure showed a poor fit for the data.

**Table 4:** The model fit results of confirmatory factor analysis.

**Factor model**	***χ^2^***	**df**	**χ^**2**^/df**	**RMSEA**	**NFI**	**GFI**	**TLI**	**CFI**	**IFI**
4-factor model	233.806	106	2.206	0.063	0.922	0.917	0.942	0.955	0.956
3-factor model	558.837	109	5.127	0.116	0.813	0.812	0.803	0.842	0.844

*χ2, Chi square value; df, degrees of freedom; χ2/df, Chi square value/degrees of freedom; RMSEA, root-mean-square error of approximation; NFI, normed fit index; GFI, goodness of fit index; TLI, Tueker-Lewis index; CFI, comparative fit index; IFI, incremental fit index*.

#### Internal Correlation Test

Among four factors of BARSHAS-SV, the correlation coefficients of four factors ranged from 0.363 to 0.640 (*p* < 0.01). The correlation coefficients between different factors and the whole BARSHAS-SV were from 0.699 to 0.864 (*p* < 0.01), as shown in Table 5.

**Table 5:** Correlation coefficients among four factors of the BARSHAS-SV and between different factors and the whole BARSHAS-SV.

**Factor**	**Factor 2**	**Factor 3**	**Factor 4**	**BARSHAS-SV**
Factor 1	0.363**	0.507**	0.425**	0.699**
Factor 2	-	0.582**	0.640**	0.808**
Factor 3	-	-	0.633**	0.864**
Factor 4	-	-	-	0.836**

Factor 1, mild respiratory system diseases; Factor 2, severe respiratory system diseases; Factor 3, respiratory system symptoms; Factor 4, treatment and recovery of respiratory system diseases; BARSHAS-SV, Brief Adolescent Respiratory System Health Assessment Scale-Student Version. Pearson correlation coefficient was used. The symbol “-” showed no such correlation coefficient. The symbol “**” indicated p < 0.01.

#### Content Validity

Based on the results obtained from six expert reviews, the content validity index (CVI) of the scale was 0.941. The CVI values of all items were from 0.667 to 1.00. The CVI values of item-1, item-2, item-3, item-4, item-5, item-7, item-8, item-9, item-10, item-11, item-13, item-14, item-15 and item-17 were 1.00. The CVI values of item-6, item-12 and item-16 were 0.667. After the research team improved the item statement expression and the item wording, six subjects reported that they could clearly comprehend the meaning of every item without any difficulty.

#### Reliability

The Cronbach's α coefficient of the BARSHAS-SV was 0.910. The Cronbach's α coefficient of factor 1 (mild respiratory system diseases) was 0.781. The Cronbach's α coefficient of factor 2 (severe respiratory system diseases) was 0.829. The Cronbach's α coefficient of factor 3 (respiratory system symptoms) was 0.835. The Cronbach's α coefficient of factor 4 (treatment and recovery of respiratory system diseases) was 0.845. The mean inter-item correlation coefficient (MIIC) value of the BARSHAS-SV was 0.384. The MIIC value of factor 1 was 0.546. The MIIC value of factor 2 was 0.501. The MIIC value of factor 3 was 0.504. The MIIC value of factor 4 was 0.581 (Table 6).

**Table 6:** The Cronbach's α coefficients and the MIIC values and of four factors and the whole BARSHAS-SV.

**Factor**	**Number of Items**	**MIIC**	**Cronbach's α**
Factor 1	3	0.546	0.781
Factor 2	5	0.501	0.829
Factor 3	5	0.504	0.835
Factor 4	4	0.581	0.845
BARSHAS-SV	17	0.384	0.910

*Factor 1, mild respiratory system diseases; Factor 2, severe respiratory system diseases; Factor 3, respiratory system symptoms; Factor 4, treatment and recovery of respiratory system diseases; BARSHAS-SV, Brief Adolescent Respiratory System Health Assessment Scale-Student Version. MIIC, mean inter-item correlation coefficient*.

## Discussion

This study provides a practical and valid measurement instrument (BARSHAS-SV). In other studies on the development of scales for the respiratory system, some scholars have also conducted some studies and explorations. For example, Campbell et al. developed a Respiratory Distress Observation Scale (RDOS) for inpatients with an average age of 72 years and unable to self-report dyspnea. The RDOS included eight observer-rated items: respiratory rate, heart rate, paradoxical breathing pattern, accessory muscle use, grunting at end-expiration, restlessness, a fearful facial display, and nasal flaring. Each item score of the RDOS ranged from 0 to 2 points, and the total score of the RDOS was the sum of the scores of all items. The RDOS had a clinical value to measure the respiratory distress and response to clinical treatment among the inpatients (17). Haimovich et al. developed the quick COVID-19 Severity Index (qCSI) tool, a 12-point scale that used three items available at the bedside: respiratory rate, nasal cannula flow rate, and minimum documented pulse oximetry. The patients were assigned to four risk strata according to the following scores: ≥10 high risk, 7–9 high-intermediate risk, 4–6 low-intermediate risk, and 0–3 low risk (18). These scales developed mainly focused on hospitalized patients with specific respiratory diseases. These scales are more suitable for assessing respiratory diseases in adults. Therefore, the BARSHAS-SV in this study specially developed for adolescents in schools is a helpful and useful tool for assessing adolescents' overall respiratory system health. In our previous study, the research team developed a Brief Adult Respiratory System Health Status Scale-Community Version (BARSHSS-CV) (19). The BARSHSS-CV was primarily used to evaluate the respiratory system health status of adults in the community, while the BARSHAS-SV developed in this study is intended for adolescents in schools. In order to make the scale better applicable to different groups of people, the research team adjusted and improved some items. For example, adults have jobs, and their work environment may be full of dust or harmful gases, which is not a concern for adolescents. Adolescents are studying at school, and they have no jobs. Therefore, researchers deleted some items about the work environment. In order to better apply the scale to students, the researchers also modified the expression of some items. For example, researchers modified the expression of item “I often cannot work, learn, or carry out outdoor activities due to respiratory system diseases” to the expression of item “I often cannot study in class due to the treatment and recovery of respiratory system diseases.” In addition, in our previous study, the results of factor analysis of BARSHSS-CV showed that the 3-dimension structure of BARSHSS-CV was more suitable among the adults in the community. However, in this study, the results of factor analysis of BARSHAS-SV showed that the 4-dimension structure of BARSHAS-SV was more suitable among the adolescents in schools. The dimensional structures of the two scales were different. The reason for the change in the dimensional structures of the two scales may be due to the differences in the growth and development status of the respiratory system, age, living habits, and health care knowledge between adolescents and adults. The BARSHAS-SV can be used as a good and brief assessment tool for evaluating the respiratory system health status of adolescents in schools. The BARSHAS-SV may help teachers in schools and medical staff in schools conveniently and quickly assess the adolescent respiratory system health status and find the main problems of the respiratory system to provide better health education and health care services. This will eventually reduce the medical burden placed on government bodies, schools, and families (20, 21).

In the research, to evaluate a hypothesized measurement model, both exploratory factor analysis and confirmatory factor analysis were conducted (22). The sample size in the research should contain at least 10 subjects to 15 subjects per variable for the factor analysis of the scale (22). The sample size of our study was large enough for the factor analysis of the BARSHAS-SV. In the exploratory factor analysis, the researchers carried out the Bartlett sphericity test and calculated the KMO value to evaluate factor analysis's suitability. The Bartlett sphericity test was significant, and the KMO value in this research was >0.6 (23). These results revealed that the data of the scale in this research were suitable for the factor analysis. The results of exploratory factor analysis indicated that 17 items loaded substantially onto four conceptually clear factors. Dimension 1 (common respiratory system diseases) in the BARSHAS-SV-II was divided into dimension 1 (mild respiratory system diseases) and dimension 2 (severe respiratory system diseases) in the final BARSHAS-SV version. The reason for the change of the dimensions was most likely because the respiratory system diseases included the mild respiratory system diseases and severe respiratory system diseases. Hence the research team measured the respiratory system diseases from two separate dimensions (mild respiratory system diseases and severe respiratory system diseases). The 4-factor model produced a more appropriate and clearer measurement of the structure of BARSHAS-SV. In the confirmatory factor analysis, the model goodness of fit was assessed by RMSEA (<0.08 acceptable), χ2/df (<3 acceptable), NFI (> 0.9 acceptable), GFI (> 0.9 acceptable), TLI (> 0.9 acceptable), CFI (> 0.9 acceptable), and IFI (> 0.9 acceptable) (16). The results of confirmatory factor analysis of BARSHAS-SV met the above evaluation criteria. The results of confirmatory factor analysis revealed that the stability and fit of 4-factor model structure of BARSHAS-SV are both satisfactory.

The content validity reveals whether items of a scale can identify the topic and content that the research team wants to measure (12). The CVI of every item of the BARSHAS-SV indicates the number of expert choices of two and three divided by the total number of experts. The total CVI value of the BARSHAS-SV is the average value of all of items' CVI values (24). The CVI values revealed that the BARSHAS-SV was able to reflect the variables that researchers intended to measure. Each item of the BARSHAS-SV was able to measure the correct content, and the BARSHAS-SV revealed a good content validity. The internal correlation test results of the BARSHAS-SV indicated that there was a certain degree of correlation between four factors of the BARSHAS-SV; moreover, there were also some differences between the four factors of the BARSHAS-SV. Therefore, the four factors of the BARSHAS-SV were able to reflect different aspects of adolescent respiratory system health status. The four factors of the BARSHAS-SV were able to comprehensively and effectively evaluate the health status of respiratory system of adolescents.

By applying the Cronbach's alpha coefficient and mean inter-item correlation coefficient, the research team could evaluate the reliability of the BARSHAS-SV (15, 25). A usual criterion for satisfactory reliability of a scale is the Cronbach's alpha coefficient of ≥ 0.70 (12). In our study, the Cronbach's alpha coefficient of the entire BARSHAS-SV was > 0.90, and the four factors of the BARSHAS-SV were all > 0.70. If the MIIC value of a scale is > 0.30, the reliability of a scale is acceptable in the study (15). In this research, the MIIC value of the whole BARSHAS-SV was > 0.30, and the MIIC values of four factors in the BARSHAS-SV were all > 0.50. Accordingly, based on the aforementioned comprehensive analysis, the BARSHAS-SV developed in this research has a satisfactory reliability.

## Limitations and Future Direction

Adolescents who participated in this study were recruited in two districts of the Baoding City in China. Therefore, the reliability and validity of the BARSHAS-SV are limited to this population. In the future, the research team should widen the scope of sampling in more cities. The BARSHAS-SV will be more widely applied and verified in more areas of the country, so that the BARSHAS-SV can be better improved and revised in the future. In addition, in the process of using the BARSHAS-SV in other different nations, further cross-cultural BARSHAS-SV improvement and cross-cultural BARSHAS-SV validation are also needed in the future. In the future, in the process of applying and validating the BARSHAS-SV among a wider range of adolescents in more other cities, researchers will recruit more new participants to further evaluate and improve the BARSHAS-SV. Researchers also hope that those scholars from other cities or countries who see this study and are interested in this scale can use the BARSHAS-SV in their cities or countries to further validate and improve the BARSHAS-SV in the future.

## Conclusion

To sum up, the research team has rigorously developed and validated the BARSHAS-SV with proven validity and reliability. A 4-factor model of BARSHAS-SV showed good psychometric indicators for the sample of Chinese adolescents. This tool may be useful for quickly evaluating the health status of the respiratory system of adolescents in schools; however, the factorial model found must be confirmed in samples with different contexts from the present study (e.g., other countries). The BARSHAS-SV can help teachers and medical staff in schools conduct targeted health interventions and provide health guidance for adolescents in schools, making them establish a long-term healthy lifestyle, which will allow for their healthy growth.

## Data Availability Statement

The raw data supporting the conclusions of this article will be made available by the authors, without undue reservation.

## Ethics Statement

The study involving human participants was reviewed and approved by Health and Family Planning Commission of Hebei (No. 20150072). The study was also approved by Medical Ethics Committee of Hebei University. Written informed consent to participate in this study was provided by the participants' legal guardian/next of kin.

## Author Contributions

LT, YG, PW, and YZhan conceived and designed the study and wrote and revised the manuscript. HD, XW, LY, DL, YZhao, and QZ collected data and analyzed data. All authors approved the final manuscript.

## Conflict of Interest

The authors declare that the research was conducted in the absence of any commercial or financial relationships that could be construed as a potential conflict of interest.

## Publisher's Note

All claims expressed in this article are solely those of the authors and do not necessarily represent those of their affiliated organizations, or those of the publisher, the editors and the reviewers. Any product that may be evaluated in this article, or claim that may be made by its manufacturer, is not guaranteed or endorsed by the publisher.

## Abbreviations

BARSHAS-SV: brief adolescent respiratory system health assessment scale-student version
EFA: exploratory factor analysis
CFA: confirmatory factor analysis
CVI: content validity index
MIIC: mean inter-item correlation coefficient
PCA: principal component analysis
KMO: Kaiser–Meyer-Olkin
RMSEA: root-mean-square error of approximation
χ2: chi square value
df: degrees of freedom
χ2/df: chi square value/degrees of freedom
NFI: normed fit index
GFI: goodness of fit index
TLI: Tueker-Lewis index
CFI: comparative fit index
IFI: incremental fit index.
